# Supplementary material for: Impact of PCV13 and PPSV23 Vaccination on Invasive Pneumococcal Disease in Adults with Treated Rheumatoid Arthritis: A Population-Based Study
Source: Microorganisms. 2024 Oct 16;12(10):2073. doi: 10.3390/microorganisms12102073 (PMC11510061; doi:10.3390/microorganisms12102073)
Supplement: Supplementary file 1 [file microorganisms-12-02073-s001.zip › microorganisms-3200605-supplementary.pdf]

Table S1. Outcome definitions.

**International Classification of Diseases (ICD)**	**Codes**
ICD-9	790.7X, 771.83, 038.2X, 038.9X, 320.1X, 320.9X, 322.9X, 421.X, 711.0X, 730.0X, 730.2X, 480.X-486.X*, 487.0X, 510.X, 511.1X, 511.9X, 041.2X
ICD-10	G00.1, A40.3, J13, B95.3, R78.81

* ICD-9 codes excluded: '480.0', '480.1', '480.2', '480.3', '480.8', '480.9', '482.0', '482.1', '482.2', ‘482.31', '482.32', '482.4', '482.40', '482.41', '482.42', '482.49', '482.80', '482.81', '482.82', '482.83', '482.84', '483.0', '483.1', '484.1', '484.3', '484.5', '484.6', '484.7', '487.0', '771.83'.
